# Integration of HIV Prevention With Sexual and Reproductive Health Services: Evidence for Contraceptive Options and HIV Outcomes Study Experience of Integrating Oral Pre-exposure HIV Prophylaxis in Family Planning Services in Lusaka, Zambia

**DOI:** 10.3389/frph.2021.684717

**Published:** 2021-07-13

**Authors:** Margaret Phiri Kasaro, Ntazana Sindano, Manze Chinyama, Mayaba Mudenda, Florence Chilaisha, Joan T. Price, Modesta Chileshe

**Affiliations:** ^1^University of North Carolina Global Projects Zambia, Lusaka, Zambia; ^2^Division of Global Women's Health, Department of Obstetrics and Gynecology, The University of North Carolina at Chapel Hill, Chapel Hill, NC, United States

**Keywords:** family planning, integration, sexual and reproductive health, oral pre-exposure prophylaxis, HIV prevention

## Abstract

The WHO guideline on the integration of family planning (FP) and pre-exposure HIV prophylaxis (PrEP) to enhance the health of women and adolescent girls is reflected in the Zambia Consolidated Guidelines for Treatment and Prevention of HIV Infection, 2020. There is however a dearth of data on the integration of PrEP and FP in Zambia. We describe the integration of oral PrEP in FP services using the Evidence for Contraceptive Options and HIV Outcomes (ECHO) study experience at Kamwala District Health Center in Lusaka, Zambia. The provision of oral PrEP at Kamwala started in October 2017, lasting for ~11 months, and utilized the model where initial processes to offer PrEP were on-site followed by off-site referral to laboratory and PrEP provider services. The characteristics of 658 women who enrolled in ECHO at Kamwala are representative of women accessing FP services in Lusaka. About 644 of the enrollees were offered oral PrEP. The proportion of women accepting PrEP was low at 1.08% and the proportion of study visits at which PrEP was requested was also low at 0.57%. Those who accepted PrEP were above 20 years old, married, with at least primary education, sexual behavior, and risk comparable to decliners. The ECHO study experience indicates that the setup and integration of oral PrEP and FP services are feasible in the setting. However, uptake of PrEP was very low. Possible contributory factors were as follows: (1) timing of introduction of PrEP midway in the study, (2) PrEP being a new intervention, (3) challenges of autonomy of young women to include a daily pill into their lives and anticipated challenges to adherence because of fear of adverse events, (4) possible underdetermined risk due to use of an unvalidated risk assessment tool and assessment by health care provider vs. self-assessment, and (5) extra layer of challenges to negotiate due to needing for off-site referrals. Following these findings, we conclude that further research through demonstration projects of integration of oral PrEP and FP may provide solutions to low uptake. This information is critical for scaling up of integration HIV prevention services and sexual and reproductive health (SRH) services.

## Introduction

African women are disproportionately affected by HIV infection. Sub-Saharan Africa carries more than 70% of the global burden of infection with women bearing the brunt of this burden. In particular, adolescent girls and young women aged 15–24 years have up to eight-fold higher incidence of HIV infection compared to their male peers (1). In Zambia, the 2,018 numbers indicate that 11.3% of adults were living with HIV out of which 14.3% are women compared to 8.8% men. Adolescent girls and young women are four times at higher risk with the prevalence of 5.7% compared to their male peers at 1.8% (2).

The need for contraceptive services in Zambia is high with a total fertility rate (TFR) of 4.7 births per woman, the contraceptive prevalence rate (CPR) standing at 50%, and unmet need of 20% for married women (3). The need for contraception is likely to be underestimated as this information excludes unmarried women. Efforts to both increase provision of contraception and reduce HIV transmission among women can be delivered in one stop. Family planning (FP) clinics provide services to women at risk for acquiring HIV and could be a vehicle for providing both contraceptive and HIV prevention services (4, 5).

The HIV prevention “toolkit” comprises both behavioral and biomedical approaches. Behavioral approaches focus on reducing high-risk practices including non-condom protected sexual encounters. Biomedical HIV prevention approaches encompass a diverse array of strategies including HIV counseling and testing, linkage and retention in HIV care (test and treat), post-exposure prophylaxis (PEP), and medical male circumcision and enhanced ARV adherence among HIV seropositive individuals (treatment as prevention) (6). A recently added tool to this arsenal is pre-exposure prophylaxis (PrEP) (7, 8). Oral PrEP is the use of oral ARV medications by HIV-uninfected individuals before HIV exposure. The efficacy of oral PrEP in studies with different populations indicates higher efficacy in studies with high adherence, 42% in MSM, 62 and 75% in discordant couples, and undetermined efficacy due to low adherence in women-only studies, Fem PrEP and VOICE (9–13). Oral PrEP is, therefore, a recommended user-controlled HIV prevention strategy that has the potential to reduce new HIV infections if delivered with high coverage and if used with sufficient adherence (6, 14, 15). Interpersonal relationships and power dynamics may present challenges for women to visit health care facilities for HIV prevention services only. FP clinic visits would be acceptable reasons, and therefore, the FP clinic could be a vehicle to deliver this HIV prevention strategy to women.

The WHO has identified populations with a background HIV incidence of >3% as being at substantial risk of HIV acquisition. Other factors considered high risk include self-assessed or partner risk and being a young woman. Therefore, WHO guidance on FP and PrEP integration are that FP and HIV prevention integration is essential if the health of women and adolescent girls is to be improved (16). The National HIV/AIDS Strategic Framework (NASF) 2017–2021 of Zambia acknowledges the potential impact of PrEP particularly as an additional option in the context of combination prevention. The NASF 2017–2021 targets the prevention of HIV infection in discordant couples and key populations. This is to be achieved by implementing evidence-informed communication and advocacy strategies to increase both healthcare provider and public awareness of PrEP without stigmatizing the intervention and its potential users, nor increasing risky sexual behavior. Further, the NASF proposes integrating PrEP into other HIV prevention programs and sexual and reproductive health (SRH) services including fertility planning services and antenatal care (17). In the 2020 Consolidated Zambia HIV guidelines, scale-up and provision of PrEP are outlined. With regards to the integration of HIV prevention and FP, it states that PrEP should be provided as part of a comprehensive package that includes contraception choices (18). There is however a dearth of data on the integration of PrEP and FP in Zambia. This, therefore, is a narrative of the integration of oral PrEP in FP services using the Evidence for Contraceptive Options and HIV Outcomes (ECHO) study experience at a district health center in Lusaka, Zambia. We will describe the step-by-step processes that were followed to make oral PrEP available in the study, summarize the characteristics of all the participants on the study, characteristics of participants who were offered PrEP, those who accepted PrEP, and finally show uptake of PrEP.

## Context

### Setting

The oral PrEP/FP integration experience of the ECHO study described here was at the research site at the Kamwala Health Center (KHC) in Lusaka urban district. KHC is one of the 23 centers in Lusaka urban that provide primary health care to a population of over 2.7 million. These facilities provide health care services including Maternal and Child Health (MCH) and HIV prevention, treatment, and care. FP services and HIV prevention are provided in MCH and antiretroviral therapy (ART) departments, respectively, with guidance to provide choices for contraception in the ART department and offer a comprehensive HIV prevention package including PrEP to women who perceive HIV risk in the MCH department. KHC was one of the sites in the ECHO trial, a large multicenter, open-label, randomized clinical trial comparing HIV incidence among women randomized to intramuscular depot medroxyprogesterone acetate (DMPA-IM), a copper intrauterine device (IUD), and a levonorgestrel (LNG) implant (19).

### Population

Lusaka is the capital city of Zambia and has a population of 2.7 million and women of the reproductive age group account for about 50% of that. KHC provides primary health services to a catchment area serving ~100,000 population. The ECHO study recruited 658 HIV-negative women aged between 18 and 45 years old seeking effective contraception. About 644 out of the 658 women were offered oral PrEP.

### Methods

The oral PrEP/FP integration process is described by a detailed account of steps taken to implement the provision of PrEP and with aid of secondary data to show characteristics of study participants, acceptors of PrEP, and uptake of PrEP. At Kamwala, PrEP was included in the HIV prevention package on October 11, 2017, after adoption as the national standard in Zambia in 2017. Risk assessment for PrEP was conducted at each visit or contact with the participant using an HIV testing counseling and risk reduction script (Data Sheet 1) was modified to include information on oral PrEP as per study SOP (Data Sheet 2).

The detailed methods of the ECHO trial have been described previously (19). Briefly, between December 2015 and September 2017, 7,829 sexually active women aged 16–35 years from four countries (Eswatini, Kenya, South Africa, and Zambia), who desired effective contraception and consented to be randomized to any of the three trial contraceptive methods were enrolled. The trial assessed HIV risk acquisition of three contraceptive methods by comparing HIV incidence among women randomized to DMPA-IM, an IUD, and an LNG implant. During the trial, the HIV prevention package provided to all women included HIV risk reduction counseling; HIV counseling and testing; sexually transmitted infection (STI) testing, treatment and partner notification of STIs; condom provision; partner HIV counseling and testing, and referral for ART in discordant couples. Oral PrEP was included in the HIV prevention package in 2016–17 following WHO recommendation in 2015. Women were followed every 3 months for a maximum of 18 months, and the study ended in October 2018. Ethics review committees provided approval for the study; written informed consent was obtained from each woman prior to commencement of study procedures.

## Results

**Figure 1:**
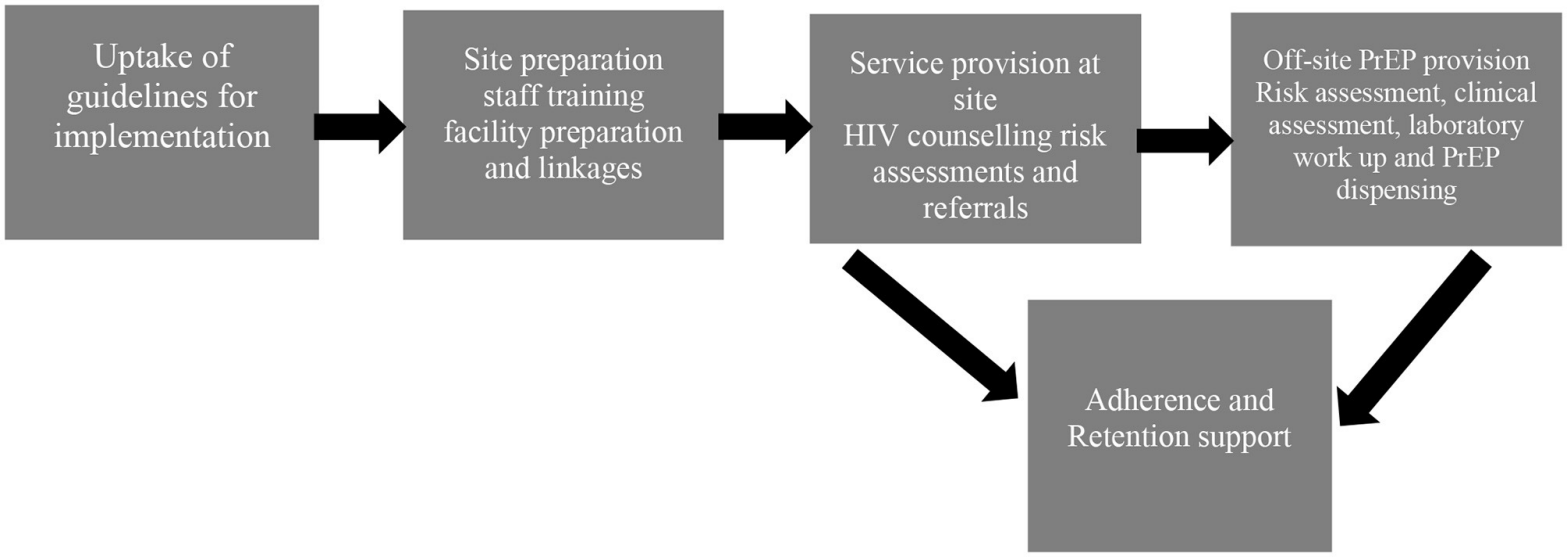
Process of implementation of the provision of Oral PrEP in the Evidence for Contraceptive Options and HIV Outcomes (ECHO) study at Kamwala.

The ECHO site at Kamwala commenced providing oral PrEP on October 11, 2017, up to the exit of the last participant in October 2018. Figure 1 is showing the activities and processes for implementation of the provision of PrEP in the ECHO study at KHC:

### Step 1

Adoption of WHO guidelines by ECHO study management team and uptake by the research site. The ECHO study management team repackaged the WHO recommendations so that evidence, rationale, and steps to implementation were used by ECHO sites successfully. The research sites were engaged regularly during this planning phase.

### Step 2

Site preparation was the next step and involved facility readiness and staff training. In this step, we took stock of the available space, structures, and resources to allow the introduction of PrEP. The existing facilities were identified and planned for dual-use if necessary. There were no extra facilities built or renovated to provide oral PrEP. Staffing needs were assessed, and it was determined that the same study staff who were conducting research procedures that included the provision of HIV prevention package would be trained to provide initial steps for oral PrEP provision. The training included content on what PrEP is, requirements for the successful provision, and addressed the attitudes of the health care provider and perceptions on the use of ART to prevent HIV.

The next part was the creation of linkages by identification and partnering with PrEP providers. The facilities for pharmacy, laboratory, and clinical staff to provide PrEP were determined to not be available and not feasible to be developed at the research site. It was, therefore, decided to utilize off-site providers. This step involved the identification of providers within the government and the private sector. Although PrEP was already part of HIV management services within the government facilities, it was not yet fully implemented. At this point, a PrEP demonstration project at a health facility about 3 km from the research site was identified. Prior to the initiation of participant referrals, there were multiple communications for introductions, sharing objectives, and establishing agreements between the research site and the PrEP demonstration project.

### Step 3

Planning for activities for adherence, safety monitoring, and retention support. The staff was trained to provide information and support so that participants would adhere to PrEP as per instructions from the off-site provider, report any adverse events and continue to take PrEP as long as they needed it.

### Step 4

PrEP provision steps at the research site and off-site. At the research site, HIV counseling, risk assessment, and referral steps in the cascade of PrEP provision were conducted. Referral to providers was made using a referral letter in a format that is used by the district health referral system. In addition, the research staff was able to access the medical records of the participants by using the existing permissions obtained from the District Health Office for the ECHO study in general. The participants were provided information on what to expect at the off-site provider i.e., risk assessment, clinical eligibility assessment, counseling for safety monitoring, dispensing of PrEP, adherence, and retention.

Table 1 shows the characteristics of all participants of the ECHO study at Kamwala, characteristics of those who were still on the study when PrEP was included in the HIV prevention package and, therefore, offered PrEP, and those who accepted PrEP. Among all ECHO participants, 97% (644/658) were offered PrEP. Over 80% of these were over 20 years old, 90% were in monogamous marriages, and 90% comprised those with primary and secondary education. In terms of sexual behavior and risk, all participants reported one current sexual partner, all reported no transactional sex, and 88% reported inconsistent use of condoms. The seven participants who accepted PrEP were all above 20 years old, married, with at least primary education, and sexual behavior and risk similar to decliners.

**Table 1:** Characteristics of evidence for contraceptive options and HIV outcomes (ECHO) participants, of those offered oral PrEP, and of those who accepted.

	**All ECHO participants (*N* = 658)**	**ECHO participants offered PrEP (*N* = 644)**	**ECHO participants accepted PrEP (*N* = 7)**
**Age**			
Over 25 years	35.41% (*N* = 233)	35.87% (*N* = 231)	42.9% (*N* = 3)
20–25 years	50.91% (*N* = 335)	50.62% (*N* = 326)	57.1% (*N* = 4)
<20 years	13.37% (*N* = 88)	13.20% (*N* = 85)	-
Unknown	0.30% (*N* = 2)	0.31% (*N* = 2)	-
**Marital status**			
Married (monogamous)	90.3% (*N* = 594)	90.53% (*N* = 583)	100% (*N* = 7)
Married (polygamous)	0.15% (*N* = 1)	0.16% (*N* = 1)	-
Divorced	0.46% (*N* = 3)	0.47% (*N* = 3)	-
Never married	7.14% (*N =* 47)	6.99% (*N =* 45)	-
Separated	1.98% (*N =* 13)	1.86% (*N =* 12)	-
**Education**			
College degree or higher	2.89% (*N =* 19)	2.8% (*N =* 18)	-
Secondary school	52.58% (*N =* 346)	52.5% (*N =* 338)	14.3% (*N =* 1)
Primary school	38.60% (*N =* 254)	39% (*N =* 251)	85.7% (*N =* 6)
No education	5.93% (*N =* 39)	5.75% (*N =* 37)	-
**Earns income**			
Yes	29 % (*N =* 191)	29.35% (*N =* 189)	42.9% (*N =* 3)
No	71% (*N =* 467)	70.65% (*N =* 455)	57.1% (*N =* 4)
**Sexual behavior assessment (past 3 months)**			
Had a sex partner (yes)	100% (*N =* 658)	100% (*N =* 644)	100% (*N =* 7)
Had new sex partner (yes)	0.15% (*N =* 1)	0.16% (*N =* 1)	-
Had new sex partner (no)	99.85% (*N =* 657)	99.84% (*N =* 643)	100% (*N =* 7)
Had sex for money (no)	100% (*N =* 658)	100% (*N =* 644)	100% (*N =* 7)
Had anal sex (no)	100% (*N =* 658)	100% (*N =* 644)	100% (*N =* 7)
**Frequency of condom use**			
Always	12% (*N =* 79)	12% (*N =* 75)	42.9% (*N =* 3)
Never	24.2% (*N =* 159)	24.4% (*N =* 157)	-
Often	6.84% (*N =* 45)	6.83% (*N =* 44)	28.6% (*N =* 2)
Rarely	12.2% (*N =* 80)	12.42% (*N =* 80)	-
Sometimes	44.8% (*N =* 295)	44.8% (*N =* 288)	28.6% (*N =* 2)
**Participant reported partner HIV Status**			
Partner HIV negative	90.12% (*N =* 593)	89.9% (*N =* 579)	28.6% (*N =* 2)
Partner HIV positive	1.37% (*N =* 9)	1.4% (*N =* 9)	71.4% (*N =* 5)
Partner HIV status unknown	8.51% (*N =* 56)	8.7% (*N =* 56)	-
**Partner has sex with others by participant report**			
Yes	10.79% (*N =* 71)	10.87% (*N =* 70)	14.3% (*N =* 1)
No	34.80% (*N =* 229)	34.63% (*N =* 223)	85.7% (*N =* 6)
Don't know	54.41% (*N =* 358)	54.41% (*N =* 358)	-

Uptake was assessed by determining the proportion of acceptors of PrEP out of all participants who were offered PrEP and the proportion of visits when PrEP was requested out of all visits after PrEP provision was implemented. Table 2 shows that PrEP uptake by participants was low at 1.08% and the proportion of study visits at which PrEP was requested was also low at 0.57%.

**Table 2:** Oral PrEP uptake.

**By proportion of participants ever accepting PrEP**	**By repeat acceptance of PrEP**
Total offered PrEP	Study visits where PrEP was offered
644 participants	2,438 visits
Participants accepting Oral PrEP	Visits with PrEP accepted
7 participants (1.08%)	14 visits (0.57%)

## Discussion

The 2020 Zambia HIV guidelines for the prevention of HIV within contraceptive context are in sync with the WHO recommendation. The ECHO study experience at KHC has shown that with available internal resources and creating linkages with external resources, integration of oral PrEP and FP is feasible in our setting. However, although the process was successfully set up, uptake of oral PrEP in the study was very low and the possible explanations include individual, community, and structural factors.

The demographic characteristics and sexual behavior of women who were offered oral PrEP in ECHO at KHC were young, married, with at least primary education, and reported sexual behavior and risk included having one current sexual partner and inconsistent use of condoms. This profile of women who participated in ECHO is similar to that of women who participated in the Zambian Demographic and Health Survey, 2018 (18).

The feasibility of integration has been demonstrated within this ECHO study experience and other studies (5, 20, 21). The first step in the implementation of PrEP at KHC is representative of strong political will. There was step-by-step support from the ECHO leadership team to the ECHO sites during the process. The implementation model that we used was to maximize available internal resources and create linkages with external providers for components of the cascade that we could not provide. The internal resources include space and staff who were already providing FP and HIV risk-reduction counseling. The research site was able to initiate processes to offer and assess risk and need for PrEP by providing the relevant training to the staff. There were no changes to the physical facilities. Linkages were identified and created with providers who could assess, provide PrEP and monitor for safety. In addition to the participant report on accessing and experience of PrEP, the agreement with the external partner allowed us to access participant medical records, and therefore, we were able to provide safety monitoring, adherence, and retention support to the participants. Other researchers have described models of integration that have a PrEP-dedicated nurse stationed in the FP clinic to lead the delivery of counseling about HIV risk and provision of PrEP. Women accessing FP services would first complete other services, including HIV testing, and then referral to the PrEP-dedicated nurse would be done. PrEP visit schedules would mirror approaches used in FP clinics (5). Lessons from the contraception world emphasize that introduction of PrEP should be strategic to include a focus on the interface between PrEP and users, method mix, and delivery methods (22). In the US, strategies to increase uptake of PrEP in SRH services have been proposed in a call for leadership in the provision of PrEP to women and these include: identifying a clinic champion to motivate and lead by example, encouragement to use the many existing resources to train staff and educate clients about PrEP, utilizing PEP transition to PrEP opportunities, coupling PrEP visits with contraceptive visits, using phone contact for visits that may not require in-person visits, and engaging the community to increase demand and knowledge of PrEP (4).

Although we were able to setup the machinery to provide oral PrEP, uptake in the ECHO study at KHC was very low, lower than any uptake recorded so far. Other researchers have shown uptake between 22% within the FP context (5) and 95% within a clinical trial setting for young women (23). Assessment of risk in both studies indicated that there were high levels of self-perceived risk of acquiring HIV because of unknown HIV status of partner, partner having other sexual partners, and inconsistent condom use (5, 23). Other factors that these studies indicate contributed to the uptake of PrEP, are the impact of community education and raising awareness of HIV prevention including PrEP done through the combined effort of study staff, community outreach teams, and advisory boards (5, 20). In the ECHO study as a whole, 622 out of 7,829 women reported using PrEP with a median duration of use at 85 days (IQR 39–96) before study exit (19).

The very low uptake could be attributed to several factors including the timing of PrEP introduction into the study, and it is a new intervention. PrEP was introduced about midway into the study after participants had “settled down” with the study procedures and interventions. This was compounded by the fact that PrEP is a new intervention. There could have been a lack of knowledge and skepticism to try the new intervention both of which could have led to low demand. However, a more compelling reason for low demand could have been because the majority of women enrolled on ECHO at KHC were young and that this age group lack gender autonomy and may be faced with challenges of incorporating a daily intervention into their lives (24, 25). Further hesitancy may have been caused by anticipated challenges of adherence such as fear of adverse reactions.

We also think that because we did not use a validated risk assessment tool that this could have led to the poor determination of risk perception. Several risk assessment tools have been developed for use with various at-risk populations including one developed and validated by Balkus et al. (26) to predict HIV acquisition among African women. The tool was based on data from trials of biomedical HIV prevention interventions (VOICE, HPTN 035, and FEM-Oral PrEP). Such tools help to accurately identify individual risk so that PrEP is offered to those who can benefit the most (27). In addition, risk assessment may also have been underdetermined due to assessment by health care workers rather than by self-assessment.

Finally, low uptake of PrEP at KHC could also be attributed to the need for off-site referral. These referrals may have presented another layer of barriers such as distance and transport costs, meeting different providers, and the need for further assessments including laboratory testing.

This work had some limitations. Although the feasibility of integrating PrEP and FP services was demonstrated, uptake was very low with only seven women accepting to use PrEP. The data presented in this study is as collected in the original study design and no further in-depth qualitative data were collected to investigate why women would decline PrEP. In addition, other measures of success of implementing PrEP in FP settings such as adherence and retention were not determined. The implementation process, successes, and challenges described here are specific to Lusaka and may not apply to different geographical settings.

This analysis joins the first few assessments of integration of oral PrEP in FP services in the setting and can be part of the evidence for conducting larger implementation projects. Following these findings, we conclude that further research through demonstration projects of integration of oral PrEP and FP is needed, and this may provide insights into low uptake. This is critical for scaling up of integration HIV prevention services including oral PrEP and SRH services.

## Data Availability Statement

The raw data supporting the conclusions of this article will be made available by the authors, without undue reservation.

## Ethics Statement

The parent study, Evidence for Contraceptive Options and HIV Outcomes (ECHO) involving human participants was reviewed and approved by University of Zambia Biomedical Research Ethics Committee and University of North Carolina Institutional Review Board. The participants provided their written informed consent to participate in this study.

## Author Contributions

MK drafted the initial manuscript. NS analyzed the data. All the authors have read the manuscript, provided critical review, and approved the final manuscript.

## Conflict of Interest

The authors declare that the research was conducted in the absence of any commercial or financial relationships that could be construed as a potential conflict of interest.
