# Supplementary material for: Integration of HIV Prevention With Sexual and Reproductive Health Services: Evidence for Contraceptive Options and HIV Outcomes Study Experience of Integrating Oral Pre-exposure HIV Prophylaxis in Family Planning Services in Lusaka, Zambia
Source: Front Reprod Health. 2021 Jul 13;3:684717. doi: 10.3389/frph.2021.684717 (PMC9580744; doi:10.3389/frph.2021.684717)
Supplement: Supplementary file 1 [file Data_Sheet_1.docx]

| **HIV Testing Counselling Worksheet** | | |
| --- | --- | --- |
| **Participant ID:** | **Visit Code:** | **Visit date:** |
| **General**   - Greet client and establish rapport - Review purpose and nature of today’s session - Emphasize confidentiality - Address any immediate issues or concerns   ________________________________________________________________________________________  ________________________________________________________________________________________  ________________________________________________________________________________________ | | |
| **HIV Education and Pre‐Test Counselling**   - Review difference between HIV and AIDS - Review modes of HIV transmission and methods of prevention - Review HIV tests to be done today and tests to be done if today’s tests indicate possible infection - Review window period and how it may affect test results - Correct any misconceptions or myths - Verify readiness for testing   ________________________________________________________________________________________  ________________________________________________________________________________________  ________________________________________________________________________________________ | | |
| **HIV Post‐Test Counselling**   - Provide and explain test results - Explain additional testing that may be required per protocol - Assess client understanding of results and next steps - Provide further information and counselling relevant to client’s test results per site SOP   ________________________________________________________________________________________  ________________________________________________________________________________________  ________________________________________________________________________________________ | | |
| **Staff Initials: _____________________________ Date: _________________________________** | | |

| **Risk Reduction Counselling Worksheet** | | | |
| --- | --- | --- | --- |
| **Participant ID:** | **Visit Code:** | | **Visit date:** |
| **Risk Assessment**   - Use open‐ended questions to assess client’s HIV risk factors - Discuss whether risk factors have changed since the last visit - Probe on factors associated with higher versus lower risk (e.g., what was different about the times when you were able to use a condom compared to times when you were not?) | | | |
| **Main Risk Factors and Barriers to Risk Reduction**  ________________________________________________________________________________________  ________________________________________________________________________________________  ________________________________________________________________________________________ | | | |
| **Risk Reduction Plan — Experience and outcomes since last visit**  ________________________________________________________________________________________  ________________________________________________________________________________________  ________________________________________________________________________________________ | | | |
| **Risk Reduction Plan — Strategies until next visit**  ________________________________________________________________________________________  ________________________________________________________________________________________  ________________________________________________________________________________________ | | | |
| **Condoms Issued:**  **Yes: No:** | | **Offer Partner Counselling and Testing:**  **Yes: No:** | |
| **Additional Notes:**  ________________________________________________________________________________________  ________________________________________________________________________________________  ________________________________________________________________________________________  ________________________________________________________________________________________  ________________________________________________________________________________________ | | | |
| **Staff Initials: _____________________________ Date: _________________________________** | | | |
