# Supplementary material for: Integration of HIV Prevention With Sexual and Reproductive Health Services: Evidence for Contraceptive Options and HIV Outcomes Study Experience of Integrating Oral Pre-exposure HIV Prophylaxis in Family Planning Services in Lusaka, Zambia
Source: Front Reprod Health. 2021 Jul 13;3:684717. doi: 10.3389/frph.2021.684717 (PMC9580744; doi:10.3389/frph.2021.684717)
Supplement: Supplementary file 2 [file Data_Sheet_2.docx]

**Purpose**

To define site specific procedures for training staff on current local pre-exposure prophylaxis (PrEP) guidelines, PrEP counseling, and as applicable, PrEP provision and referrals.

**Scope**

This procedure applies to all ECHO staff involved in counseling and provision of or referral for PrEP.

**Responsibilities**

ECHO Research nurses. OAOC nurse and Study Coordinator to provide clinical care and PrEP counseling are responsible for understanding and following this SOP.

ECHO Study Coordinator or designee is responsible for training study staff in accordance with this SOP.

ECHO Site Investigator of Record has ultimate responsibility for ensuring that all applicable ECHO staff members follow this SOP.

**Background**

I. The ECHO Consortium is committed to the provision of highest standards of HIV prevention for all participants. ECHO study participants will be encouraged to consider all HIV prevention tools available to them, including use of pre-exposure prophylaxis (PrEP).

1. Partici pants in ECHO will receive risk reduction counselling, condoms, education about the efficacy of PrEP in women at risk for HIV, and, as appropriate, referrals to local providers for PrEP in collaboration with local regulatory partners if available in country.
2. Per ECHO protocol, PrEP is permitted if approved in country, available, and provided to participants by a healthcare provider at the study clinic or by external healthcare provide rs.
3. Participants will be counselled that if they so choose, PrEP can be used concurrently with their randomized contraceptive method, or any other method if they disco ntinue their randomized method , and disclosure of use is encouraged. If used , PrEP use should be documented as a concomitant medication.
4. Should national guidelines and local standard of care support PrEP, sites are encouraged to consider making PrEP available on-site. However, ECHO protocol funding cannot be used for on-site PrEP provision. PrEP will not be delivered in iso lation , but as part of a comprehensive HIV prevention package.

UNC Kamwala

# PrEP SOP

Version I.0, approved on 19 Sep, 17

**Procedures**

1. Current National PrEP-specific Guidelines / Policies
   1. Information on current local PrEP guidelines and policies is as per attached Zambia Consolidated Guidelines for Treatment and Prevention of HIV infection, 2016 page 26
2. Information for pa11icipants
   1. Messages and information provided as part of risk reduction counseling

1. The site will provide comprehensive HIV prevention package information

which will include information on oral P rEP.

11. Oral PrEP is the use of antiretroviral (ARY) drugs before HIV exposure by people who are not infected with HIV to block the acquisition of HIV. WHO recommends oral PrEP containing TDF should be offered as an additional prevention choice for people at substantial risk of HIV infection as part of combination HIV prevention approaches. Guidelines in Zambia on PrEP counseling and provision are available and disseminated in the Zambia Consolidated Guidelines for Treatment and Prevention of HIV infection, 2016 but full implementation on where to access PrEP has not yet been done.

- - 1. Eligibility for PrEP
       1. Pa11icipants not infected with HIV
       2. Oral PrEP should be initiated to negative partner in sero-discordant relationship whose **HIV** positive partner has not or recently initiated

cART.

- - - 1. Persons engaged in high risk activities such as sex work may also be considered for PrEP.
      2. Eligibility is determined by the PrEP provider.
  1. Information about PrEP co-use with contraceptive methods

1. The site will inform participants that PrEP can be used concurrently with any contraceptive method therefore can be with the methods being used in ECHO

- 1. Procedures and resources for referral for PrE P services

1. All HIV uninfected ECHO study participants will receive counseling regarding PrEP and other HIV prevention strategies. ECHO sero-converters in HIV sero-discordant relationships may also benefit from counseling regarding PrEP. The site will offer referral services for participants who wish to access PrEP to the University of Maryland supported Lusaka Urban District Health Management Team (LUDHMT) clinics which are providing as part of the integrated health care services **HIV** prevention package including PrEP These are the Railway clinic and Evelyn Hone College clinic. The site will refer using a referral letter to these centers using the LUDHMT referra l system already in place. The site will inform ECHO participants of other resources for referral for PrEP as they become available. Eligibility for PrEP is determined by the PrEP provider.

# UNC Kamwala

PrEP SOP

# Version1.0, approved on I 9 Se p, 17

ii. The site will not provide PrEP but the clinicians will provide counse lling , adherence support, and information that ongoing safety monitoring and management will be provided by the PrEP provider. If an ECHO participant on PrEP is identified as a seroconverter or possible seroconverter at an ECHO study visit, the site team with the partici pant' s consent will work with the PrEP provider to withhold the participant's PrEP.

111. Pregnant and breastfeeding women may be considered for PrEP if indicated according to current national guidelines.

- 1. Staff Training and certification

Initial training will be as per this SOP and ongoing reference to the SOP will be available for all members of staff

Manze Chinyama SC Author, Author's **Title**

tnC0L Lvv'- )0 *l-r6/*

Signature *' 7*

19 Sep, 17 Date

Margaret P. Kasaro c&---c, *>* , ;?

Reviewer, Reviewer's Title Signature

19 Sep, 17 Date

UNC Kamwala

PrEP SOP

Version1.0, approved on 19 Sep, 17
